# Hip arthroscopy and osteoarthritis: Where are the limits and indications?

**DOI:** 10.1051/sicotj/2015027

**Published:** 2015-10-16

**Authors:** Claudio Mella, Ignacio E. Villalón, Álvaro Núñez, Daniel Paccot, Claudio Díaz-Ledezma

**Affiliations:** 1 Facultad de Medicina Clínica Alemana de Santiago – Universidad del Desarrollo Santiago Chile

**Keywords:** Hip, Arthroscopy, Indication, Osteoarthritis

## Abstract

The use of hip arthroscopy, as a surgical technique, has increased significantly over the past ten years. The procedure has shown good and excellent results in symptom relief and function improvement for patients with femoro-acetabular impingement (FAI) and concurrent chondro-labral lesions. It is also a reliable method to correct the characteristic pathomorphologic alteration of FAI. However, surgical results are less successful among patients with advanced articular damage and secondary hip osteoarthritis. The aim of this article is to present some clinical and imagenological tools to discriminate the good candidates for arthroscopic FAI treatment from those who are not, due to extensive articular damage.

## Introduction

In the last 20 years, hip arthroscopy has achieved important breakthroughs, making it a safe and precise surgical technique. Despite these achievements, arthroscopy indications are still unclear and need to be defined. “Hip Osteoarthritis” is an example of a controversial indication of this technique. The reason behind this is that some morphological deformities of the bone (osteophytes) with a variable amount of chondral damage cannot be repaired satisfactorily during hip arthroscopy (Table 1). Hip arthroscopy performed in patients with advanced stages of osteoarthritis, despite having been commonly performed, is associated with poor results [1]. It may lead to a transitory and unpredictable relief of symptoms but cannot change progression of the degenerative disease when the arthroscopic treatment is done in advanced stages [2, 3].

**Table 1.**
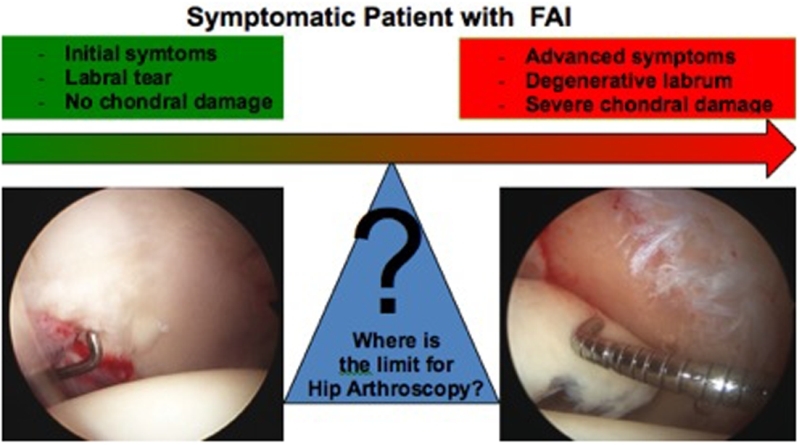
In patients with a bone deformity of the hip (PFA, dysplasia) progressive damage of the intra-articular structures (labrum, articular cartilage) occurs. In early stages, labrum and chondrolabral junction injury are present. In more advanced stages, extensive full-thickness chondral damage occurring in the weight-bearing area of the joint can be identified. To establish the limit between these two stages recognizing which patients are going to profit from arthroscopy (stabilization of chondral injury, labral repair, and correction FAI) remains a challenge in clinical practice.

Hip osteoarthritis in people under 50 years is caused in most cases by anatomical deformities such as femoroacetabular impingement (FAI) or dysplasia. Other causes, much rare, are post-trauma, rheumatic diseases, avascular necrosis, among others [4–8].

In regard to FAI, this anatomic deformity can be defined as an acetabular deformity (type “pincer”) and/or femoral deformity (type “cam”). They cause a space conflict in certain ranges of motion (specially in flexion, adduction, and internal rotation) leading to a progressive damage to the acetabular labrum and the adjacent chondral surface [9], which may progress to advanced hip osteoarthritis [10–15].

Regarding the clinical management of FAI, patients who do not have a radiographic evidence of joint damage can be easily distinguished from those with an advanced disease. While hip arthroscopy is an appropriate indication for the first group it is inadequate for the second one. However, many patients, for example, patients with high sport expectation, have intermediate stages of joint disease with early signs of osteoarthritis on X-ray or magnetic resonance imaging (MRI). In these cases, the surgeon must balance the risk and benefits to perform a hip arthroscopy (Table 1).

## Progressive chondral damage

**Figure 1.**
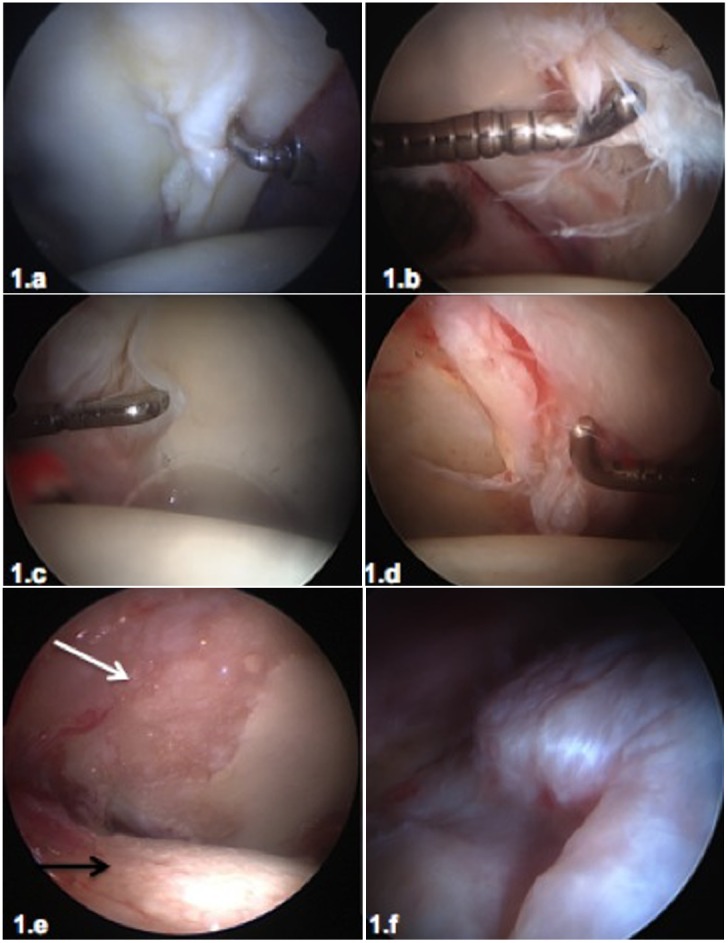
(1a–1d) Overview of arthroscopic progression of chondral damage. In early stages of joint damage, a chondro-labral instability (chondromalacia, 1a) occurs in the anterolateral part of the acetabulum. The impact of the femoral bump leads to a disruption at the chondro-labral union (1b) or to degenerative lesions of the labrum. With the progression of the joint damage, chondral flaps were created toward the central area of the acetabulum (1c and 1d). Until these flaps do not reach the load-bearing surface, these damages can be considered early stages of osteoarthritis and satisfactory outcomes with hip arthroscopy can be expected. (1e and 1f) More advanced stages of osteoarthritis lead to thinning and ulceration of the cartilage in the acetabulum load-bearing surface (1e, white arrow), progression of degenerative cartilage damage of the femoral head (1e black arrow), and formation of osteophytes (1f). These advanced lesions should be considered beyond effective treatment with hip arthroscopy.

The literature reports a wide range of options to classify the articular chondral damage based on the depth, location, and extension of the defect [16, 17]. Arthroscopy has allowed a better understanding of the physiopathology of the chondral damage in osteoarthritis of the hip. The damage secondary to FAI or dysplasia usually begins in the peripheral portion of the anterolateral region of the acetabulum. In the FAI, this is the anatomic location where the mechanical overload or impact by the femoral head occurs. In the “pincer” type of deformity, there is a direct impact on the labrum that causes an extensive degeneration of the labrum and the adjacent chondral surface. In the “cam” type of deformity, the impact leads to a chondrolabral disruption and a progressive chondral delamination. When this damage is limited to the labrum, the chondro-labral union or a peripheral chondral delamination not reaching the acetabular load-bearing surface, it can be cataloged as initial damage and suitable to be treated by hip arthroscopy (Figures 1a–1d). As the disease progresses, chondral damage spreads into the acetabular load-bearing surface, leading to cartilage delamination, full thickness chondral defects, diffuse thinning of the cartilage, development of osteophytes as well as a progressive damage of the chondral surface of the femoral head (Figures 1e and 1f). In these stages of chondral damage, arthroscopic treatment will not be advised.

## Clinical and imaging assessment

Several criteria can be used to assess whether the patient will benefit or not from a hip arthroscopy (Table 2). These can be categorized into clinical (patient history, physical examination), imaging (radiography, computed tomography or magnetic resonance imaging), or general condition of the patient (age, etiology of the disease).

**Table 2.** Clinical history, physical examination, and imaging studies (X-rays, MRI) in patients with osteoarthritis of the hip secondary to FAI or dysplasia. Preoperative evaluation of these parameters can help to quantify the degree of chondral injury or osteoarthritis in this group of patients. These clinical parameters of easy access facilitate the identification of patients in early stages, which can benefit by hip arthroscopy.

	PFA/displasia	Hip osteoarthritis
Medical history	Inguinal pain related to movement: flexion and rotation. Frequent related to sports activities	Diffuse pain (groin, lateral, buttock, thighs), permanent, by walking and weight bearing. Night and rest pain
Physical examination	Normal gait. Restriction only in flexion and 90° rotation (FADIR, FABER)	Claudication, multidirectional restriction of range of movement
Radiology	Signs of FAI or Dysplasia, without joint space narrowing	Joint space narrowing (<2 mm). Osteophytes
Magnetic resonance (MR)	Labral tear, damage in chondrolabral junction	Chondral damage in load bearing area and femoral head. Bone edema, subchondral cysts.
Treatment option	Consider hip arthroscopy	Hip arthroscopy not recommended

### Medical history

Medical history will be essential to analyze in detail the evolution and characteristics of the patient’s pain. Pain secondary to initial osteoarthritis (labrum injury, peripheral chondral lesion) is related to the movement, especially flexion and rotation, and is usually located in the groin, buttock, or thighs. On the other hand, in advanced osteoarthritis (extended chondral injury in weight-bearing zone, osteophytes), pain will be more permanent, present during loading and rest, more diffusely located around the hip, especially in the gluteal region. Pain with these features indicates more advanced chondral damage and the patient will likely be beyond the limit of an effective arthroscopic treatment. Other causes of pain that radiate to the hip must always be ruled out, for example, lumbar degenerative pathology among others [13, 18].

### Physical examination

It will be essential to assess the gait, the ranges of motion, and specific clinical signs that provoke pain. In the early stages of FAI the patient will have a normal gait and the range of motion will be limited only in the classical impingement manoeuvers. Pain can be elicited in 90° flexion, internal rotation, and adduction (anterior impingement manoeuver, FADIR). Pain triggered by these manoeuvers can be a sign of a labrum injury or peripheral chondral damage. External rotation and abduction in 90° flexion (FABER) can also be limited and will induce pain in FAI.

On the contrary, in more advanced stages of osteoarthritis, patients might present with claudication during the gait assessment, a multidirectional reduction of the range of motion and pain will be elicited in the forced movements in all axes. These signs are the manifestations of an advanced chondral damage and probable development of marginal osteophytes, which cannot be addressed effectively by hip arthroscopy.

### Radiology

**Figure 2.**
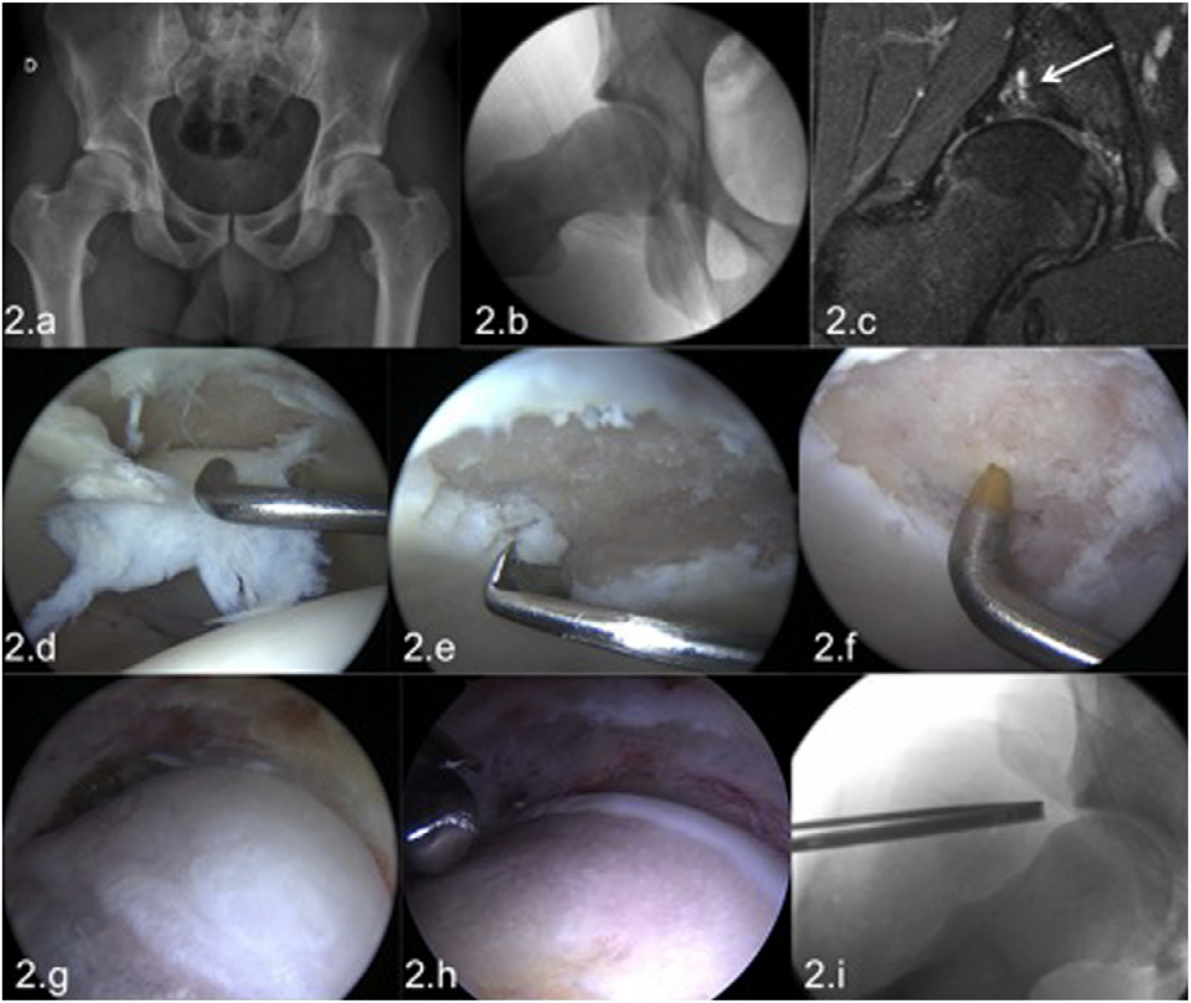
Male patient, 23 years old, medical student and amateur rugby player with sports-related pain in the right hip. The radiology demonstrated a FAI with a significant CAM deformity without significant joint space narrowing (2a, 2b). MRI showed a labral injury, a focal chondral lesion with subchondral cysts, and subchondral edema (2c). In spite of these ominous imaging signs given his young age and no other signs of osteoarthritis, hip arthroscopy was performed. At the arthroscopy an extensive full-thickness chondral lesion was found in the load-bearing surface of the acetabulum (2d); no femoral head cartilage lesions were present. The chondral lesion was treated by abrasive chondroplasty (2e) and microfractures (2f). In the peripheral compartment the extensive femoral bump (2g) was resected (2h). Intraoperative dynamic testing at the end of the femoroplasty demonstrated absence of impingement; the axial radiography demonstrated a satisfactory correction of the cam deformity (2i). Nevertheless after this arthroscopic repair, the future of this joint is uncertain with a high risk to progress to osteoarthritis in this young patient.

Accurate radiographic study is a must as it will be the basis not only for the diagnosis and the assessment of the bone deformity, but also to assess the severity of the osteoarthritis. An anteroposterior pelvic view (AP) [19], an axial radiograph of the femoral neck (true axial, Dunn projection [20]), and a false profile [21] must be performed. In the early stages of the disease, the typical radiological signs of underlying disease (dysplasia, FAI type cam or pincer) will be present, without a joint space narrowing or osteophytes (Figure 2).

In more advanced stages of osteoarthritis, joint space narrowing less than 2 mm will be evident and is considered a poor prognosis indicator [22, 23]. A posterior joint space narrowing and osteophyte formation in both the acetabular rim and the femoral neck in the false-profile imaging can also be recognized.

Joint space less than 2 mm, and the presence of osteophytes, are indirect radiological signs of advanced chondral damage. These features mark the limit for effective arthroscopic treatment. The cutoff point, measured in the load-bearing surface, has been shown to be a reliable parameter to define hip osteoarthritis with greater reproducibility than other measures [24–27].

### Magnetic resonance

**Figure 3.**
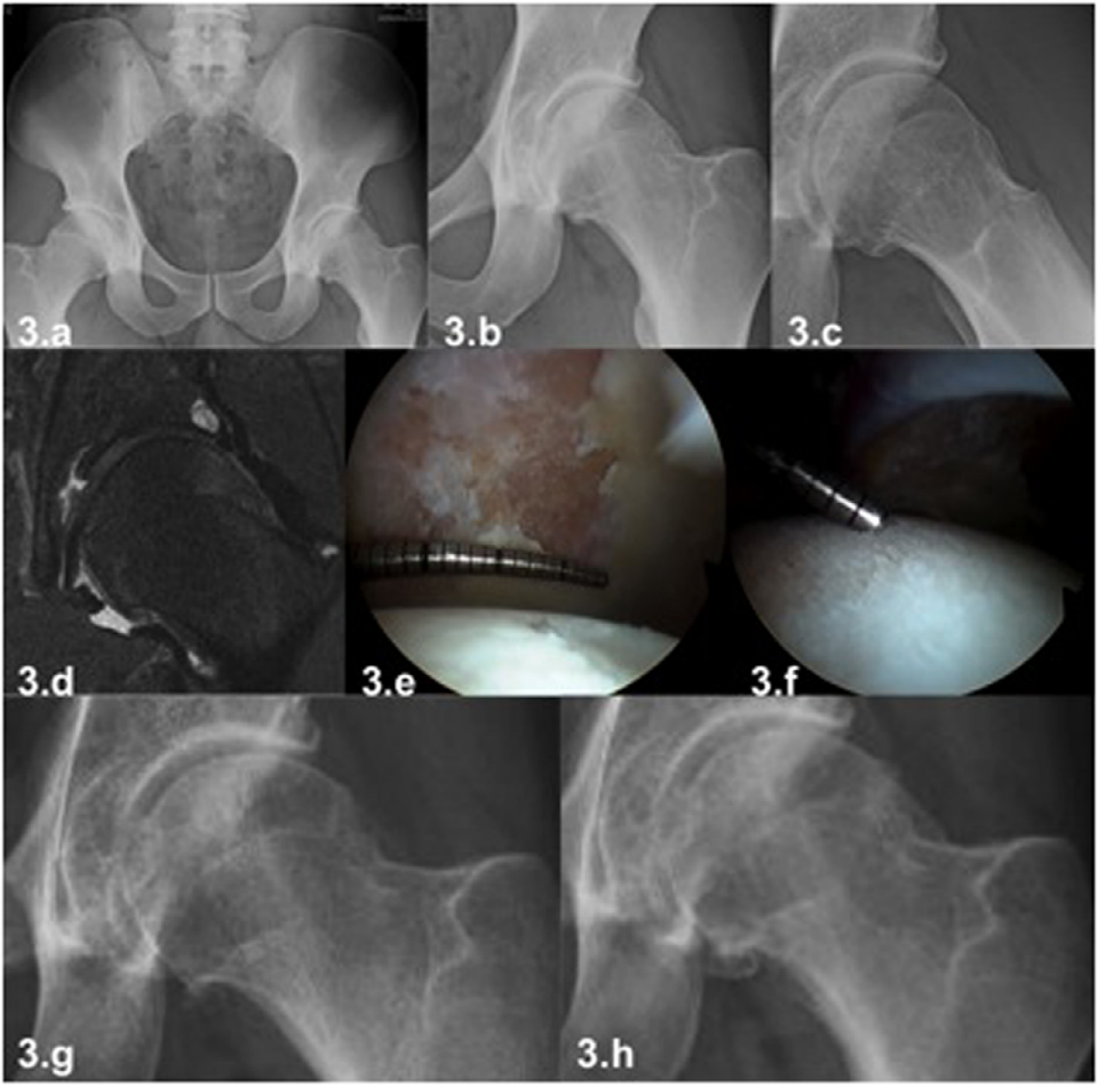
Male patient, age 32 years, intensive recreational athlete (triathlon, mountain climbing) with left hip pain in flexion and rotational movements. Clinical examination demonstrates a restriction of internal rotation in flexion above 90° associated with pain (anterior impingement). The radiograph demonstrates a FAI with a cam deformity and joint space narrowing in the peripheral joint area (3a–3c). The MRI showed a chondrolabral lesion and acetabular subchondral cyst (3d). Considering his young age and his rejection to a THR a hip arthroscopy was performed. Full-thickness chondral damage in acetabulum (3e) and diffuse fibrillation of cartilage in femoral head was found during the arthroscopy (3f). These should be considered an advanced osteoarthritis finding with a very unpredictable clinical outcome. The radiography one year later shows the progression of the chondral damage of the hip (3g). This case shows that with hip arthroscopy a temporary relief of pain can be achieved in cases of advanced chondral damage but it cannot change the course of the disease and progression to osteoarthritis of the hip.

In early stages of osteoarthritis, the MRI can show acetabular labrum abnormalities (rupture, partial or full detachments, degenerative changes) and chondral damage near the chondrolabral union. In advanced stages of osteoarthritis, chondral damage will extend into the central and load-bearing areas in the acetabulum with variable thickness (delamination, chondral ulcers, diffuse thinning). Femoral head chondral damage will also appear, characterized by progressive defibrillation until a diffuse chondral ulcer occurs in the most advanced stage. Some indirect signs of severe chondral damage can be detected on MRI like bone edema in loading area (either acetabular or femoral) and the presence of subchondral cysts of varying sizes. These signs can be considered of poor prognosis for a treatment based on hip arthroscopy (Figure 3) [28].

Actually new technology is available such as T2 mapping [29], dGEMRIC [30], using scores systems to grade the degree of chondral damage [31] or evaluate the femoral version [32]. While promising options are considered, their usefulness has not yet been fully demonstrated and is not the “state of the art” today.

### Computed tomography (CT) scan

During the early stages of the disease, CT scan will not show alterations beyond the existing bone deformity, however, in advanced osteoarthritis, it will evidence the presence and location of osteophytes, the presence of subchondral cysts in the acetabulum, and the narrowing of the joint space. Joint space narrowing can be documented in the most anterior or posterior hip region, which is more difficult to be recognized in conventional radiology. A recent study reports the utility of three-dimensional reconstructions of CT scan to predict intra-articular arthroscopic findings [33].

Independent from the clinical findings and the imaging studies, other factors such as the etiology of osteoarthritis, age and the patient’s expectations must be considered.

## Osteoarthritis etiology

There is still no evidence that hip arthroscopy can positively influence long-term course of hip osteoarthritis. However, cam deformity correction and partial resection of the acetabular chondral damaged area (pincer) could restrict and protect the joint from further chondral damage. The clinical benefit of these procedures must be confirmed prospectively, with the assessment of long-term outcomes of treated patients. The evidence sustains that cam deformity (represented by an increased alpha angle) is an independent predictor of total hip replacement (THR) in patients without arthritis followed for 20 years [34].

While this is true for FAI, it cannot be extrapolated to dysplasia, as no correction of the etiological factor is made with arthroscopic intervention. Moreover, arthroscopy can increase joint instability (capsulotomy, any partial resection of labrum, etc.) [35] leading to accelerated joint deterioration [36]. Due to this, arthroscopy in a patient with hip dysplasia and chondral damage should be carefully selected, since there is no evidence of a positive clinical result [37].

### Age and patient expectations

**Figure 4.**
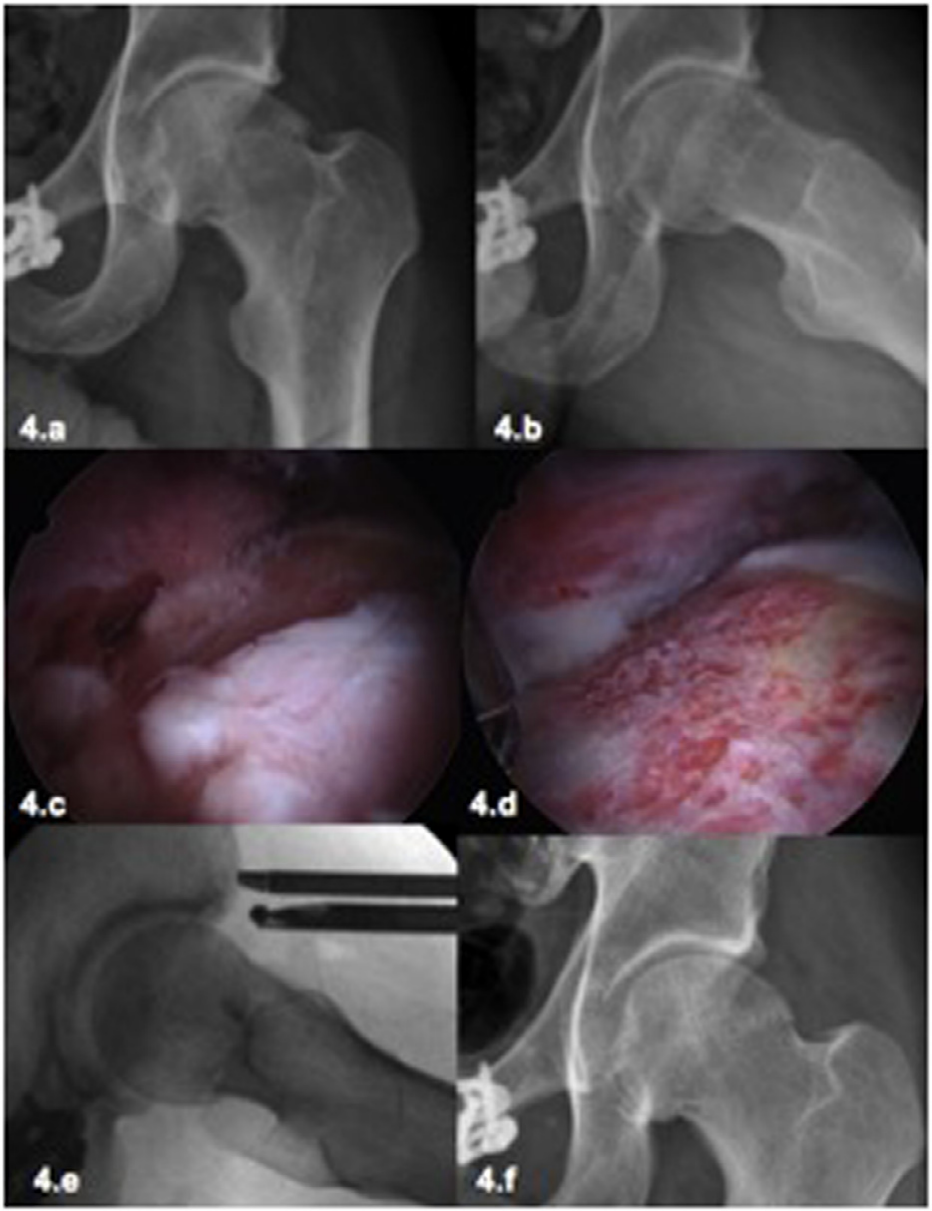
Male patient, 32-year old physician, intensive recreational sports (Karate), 4 years after left hip arthroscopy for FAI with persistent pain and restriction of movement (ROM). Despite the evident radiological signs of osteoarthritis (4a, 4b) the patient insisted on a repeat arthroscopy due to the pain and limitations for sports. Considering the young age and the conserved joint space in the load-bearing area, a revision arthroscopy was performed. During the hip arthroscopy the chondrolabral damage was treated in the central compartment. In the peripheral compartment the osteophytes were identified and resected (4c) as well as the cam deformity. A satisfactory correction of the deformity was achieved (4d–4f). Even in this case with a satisfactory anatomic correction and a satisfactory clinical short-term result, the long-term outcome is still unknown with a high risk of progression to osteoarthritis.

There is no age limit for hip arthroscopy, as positive results have been proven in young and old patients [38]. Overall, in patients under 40 years, hip arthroscopy can be indicated treating larger chondral defects in an attempt to prolong the lifetime of the hip and eventually prevent THR (Figures 2 and 4).

For patients older than 50 years (premature joint failure age cutoff in clinical trials [6]), indications for arthroscopic procedures should be more restricted as advanced chondral damage is usually present and total hip replacement (THR) in this age group has also excellent functional results with good long-term survival of the implant in most of the cases [39].

**Figure 5.**
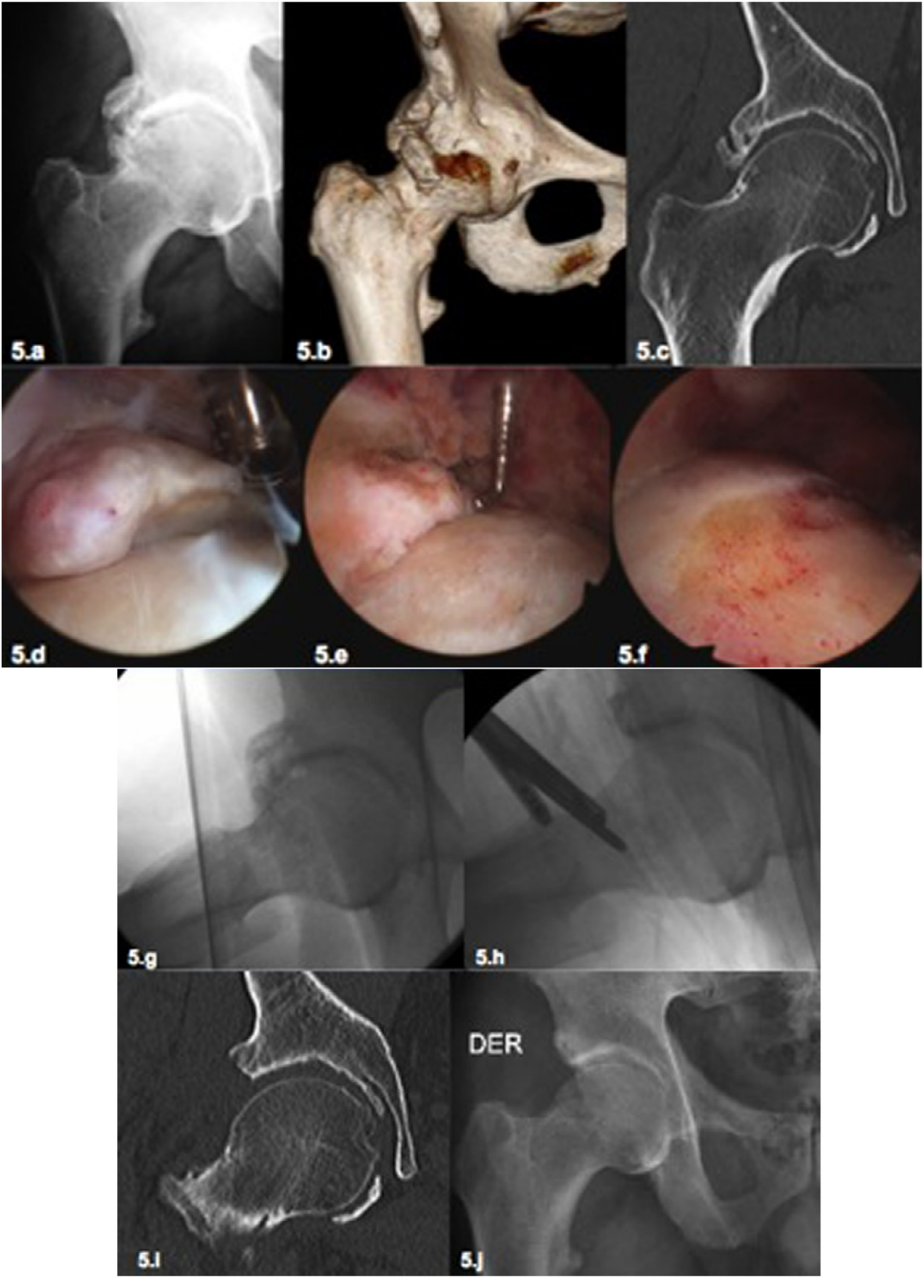
(5a–5f) Male patient, 65 years with an advanced osteoarthritis of the hip with a significant ROM (5a). The patient and his wife (orthopedic surgeon) refused the proposed treatment with a total joint replacement insisting to perform a hip arthroscopy. The complemental imaging studies with CAT Scan demonstrate the extensive osteophytes (5b) with conservation of the joint space in the load-bearing area (5c). After a long discussion with the patient without creating false expectations, a hip arthroscopy was performed. The osteophytes in the peripheral compartment were resected and the acetabular labrum was debrided (5d, 5e). The acetabular rim was resected as well as the existing cam deformity (5f). (5g–5j) The same patient from Figures 5a–5f. The intraoperative radioscopy demonstrated the satisfactory resection of the osteophytes and the bone deformity (5g, 5h). The postoperative CAT scan (51) and the postoperative X-ray (5j) demonstrated also a satisfactory result. Five years after this surgery the patient has still an excellent clinical outcome (HHS 92) without progression of osteoarthritis. This case demonstrates that we still do not know where exactly are the limits for hip arthroscopy in cases with osteoarthritis. It demonstrated also that in advanced pincer cases with arthritis due to the resection of the deformity, good results can be achieved (5i, 5j). Independent of this satisfactory clinical result, in cases like these a total joint replacement (TJR) is the most effective and predictable option of treatment but it was refused by the patient.

Besides age, patient’s expectations must always be considered and clarified before the procedure. Patients with high sports expectation usually reject THR as it can lead to restrictions in physical activity. In these cases, a frank and detailed discussion with the patient is necessary to discuss the risks and benefits of hip arthroscopy (Figure 4). The risk of poor functional outcome in cases of advanced chondral damage should be stressed, without raising false expectations and confronting the patient joint damage reality (Figure 5) [40].

## Treatment options in hip arthroscopy

When hip arthroscopy is performed in advanced stages of arthritis, significant degenerative damage to the labrum and to the articular cartilage can be found as well as to osteophytes of diverse magnitude (Figures 4 and 5).

The labrum will usually be degenerated or calcified and arthroscopic repair will not be an effective treatment. Moreover, keeping an unstable portion of the labrum risks persistent painful symptoms. Therefore the debridement of the damaged labrum is recommended in selected cases. This can lead to higher reoperation rates and lower outcomes [41]. Labral reconstruction with a graft is not an effective option in patients with advanced chondral damage.

Unstable chondral lesions should be managed by an abrasive chondroplasty resecting unstable fragments. Performing micro fractures is only indicated in focal lesions and not in cases of diffuse chondral damage or of the acetabulum or the femoral head (Figure 2).

Osteophytes in the rim of the acetabular fossa can be resected, ideally using a smaller and curved burr. However, the clinical effectiveness of this resection is uncertain. In the peripheral compartment it is also possible to resect osteophytes at the junction of femoral head and neck especially in the medial and lateral regions (Figure 4). In the case of large osteophytes in the medial region, it may be useful to use an anterior accessory portal during arthroscopy with a more direct approach to the region.

In summary, in cases of more advanced stages of hip osteoarthritis only a few arthroscopic therapeutic options with an uncertain clinical effectiveness are available. The arthroscopic treatment may provide temporary relief to reduce patient discomfort without changing the natural course of the disease.

## Discussion

It is widely accepted that bone deformities of the hip as FAI and dysplasia, among others, can cause a progressive chondral damage leading to osteoarthritis.

Hip arthroscopy has been developed as a treatment option for patients in early stages of the disease. It can effectively treat femoral and acetabular deformities (cam and pincer, respectively), and repair injuries of the acetabular labrum and adjacent cartilage.

The different options to repair articular cartilage are still limited especially when these are more extensive and deep, and when located in the load-bearing surface or femoral head. Hip arthroscopy, performed in patients with advance osteoarthritis, has poor clinical outcomes. In 2011, McCarthy et al. [42] presented his experience of 10 years of follow-up after hip arthroscopy reporting a 67% success rate in 111 hips. He describes as a poor predictor of results being elderly and having advanced chondral lesions (Outerbridge 4 type). Haviv and O’Donnell [43] reported their experience in 564 hips with osteoarthritis stages between Tönnis 1 and 3. They conclude that 50% of patients who were treated with arthroscopy had to have a THR on average 1.5 years later. The factors considered to be of poor arthroscopy prognosis described in this study are: patient’s age (patient older than 55 years) and advanced osteoarthritis (Tönnis 3).

Horisberger et al. [44] state their experience of 20 patients with type 2 or greater Outerbridge chondral lesions in the load-bearing surface or femoral head treated by hip arthroscopy. Fifty percent of these patients progressed to a THR within 3 years of the procedure. They also conclude that critical risk factor for negative outcome is the presence of osteoarthritis Tönnis stage 3 as well as chondral lesions in the femoral head.

Byrd and Jones [45] described that after following the evolution of five athletes who had hip arthroscopy performed in advanced stages of osteoarthritis, all of the patients needed THR. They described as factors of poor prognosis: sclerosis, subchondral lesions, and the presence of osteophytes.

Philippon et al. [8] published in 2009 his experience in 112 cases of hip arthroscopy performed in patients with FAI; ten of them required THR. He described as the main risk factors for poor arthroscopy prognosis: advanced age, joint space narrowing less than 2 mm, chondral lesions in femoral head, and resection of acetabular labrum. Lately he presented his experience in patients older than 50 years, showing that 20% of the patients required THR. In patients with joint space <2 mm, the survival of hip arthroscopy at 3 years was 57% [22].

Beck et al. [46] in their series of surgical hip dislocation also showed that age over 50 years is a poor prognostic factor in addition to the existence of a “coutre-coup” injury in the posterior acetabulum with a translation of the femoral head secondary to anterior over coverage.

The evidence review shows that the poor prognostic factors for hip arthroscopy are as follows: (1) patients operated after the fifth decade of life, (2) advanced chondral damage (Outerbridge 2 or greater) in the weight-bearing surface, (3) degree of radiological osteoarthritis Tönnis 3 or higher, (4) joint space narrowing less than 2 mm, and (5) cases where the MRI show chondral lesions in the femoral head, subchondral cysts, subchondral edema, or a posterior translation of the femoral head [47].

In our experience, hip arthroscopy can obtain good results when cartilage damage is limited to the periphery of the acetabulum (chondrolabral union, peripheral chondral defect). The results will not be promising if chondral defects are present in the loading area of the acetabulum, degenerative changes in femoral head, or presence of extensive osteophytes.

## Final thoughts

Variable degrees of joint damage are often present in patients with bone deformity who are at risk of hip osteoarthritis (PFA, dysplasia). It is actually hard to predict the real degree of articular damage, despite the tools available at present. This process is of paramount importance for the clinical management of this group of patients determining the most adequate treatment option (nonsurgical treatment, arthroscopic hip surgery, or joint replacement). In this review some clinical and image studies that are helpful to make a sound decision in this patient group were presented.

## Conflict of interest

The authors declare no conflict of interest in relation with this paper.
